# Mechanical high-intensity focused ultrasound creates unique tumor debris enhancing dendritic cell-induced T cell activation

**DOI:** 10.3389/fimmu.2022.1038347

**Published:** 2022-12-07

**Authors:** Renske J. E. van den Bijgaart, Vera E. Mekers, Fabian Schuurmans, Tonke K. Raaijmakers, Melissa Wassink, Andor Veltien, Erik Dumont, Arend Heerschap, Jurgen J. Fütterer, Gosse J. Adema

**Affiliations:** ^1^ Radiotherapy & OncoImmunology Laboratory, Department of Radiation Oncology, Radboud University Medical Center, Nijmegen, Netherlands; ^2^ Department of Medical Imaging, Radboud University Medical Center, Nijmegen, Netherlands; ^3^ Image Guided Therapy, Pessac, France; ^4^ Department of Robotics and Mechatronics, University of Twente, Enschede, Netherlands

**Keywords:** high-intensity focused ultrasound (HIFU), tumor antigens, dendritic cells, TLR9 agonist CpG, cancer immunotherapy

## Abstract

**Introduction:**

*In situ* tumor ablation releases a unique repertoire of antigens from a heterogeneous population of tumor cells. High-intensity focused ultrasound (HIFU) is a completely noninvasive ablation therapy that can be used to ablate tumors either by heating (thermal (T)-HIFU) or by mechanical disruption (mechanical (M)-HIFU). How different HIFU ablation techniques compare with respect to their antigen release profile, their activation of responder T cells, and their ability to synergize with immune stimuli remains to be elucidated.

**Methods and results:**

Here, we compare the immunomodulatory effects of T-HIFU and M-HIFU ablation with or without the TLR9 agonist CpG in the ovalbumin-expressing lymphoma model EG7. M-HIFU ablation alone, but much less so T-HIFU, significantly increased dendritic cell (DC) activation in draining lymph nodes (LNs). Administration of CpG following T- or M-HIFU ablation increased DC activation in draining LNs to a similar extend. Interestingly, *ex vivo* co-cultures of draining LN suspensions from HIFU plus CpG treated mice with CD8^+^ OT-I T cells demonstrate that LN cells from M-HIFU treated mice most potently induced OT-I proliferation. To delineate the mechanism for the enhanced anti-tumor immune response induced by M-HIFU, we characterized the RNA, DNA and protein content of tumor debris generated by both HIFU methods. M-HIFU induced a uniquely altered RNA, DNA and protein profile, all showing clear signs of fragmentation, whereas T-HIFU did not. Moreover, western blot analysis showed decreased levels of the immunosuppressive cytokines IL-10 and TGF-β in M-HIFU generated tumor debris compared to untreated tumor tissue or T-HIFU.

**Conclusion:**

Collectively, these results imply that M-HIFU induces a unique context of the ablated tumor material, enhancing DC-mediated T cell responses when combined with CpG.

## Introduction

In interventional oncology, *in situ* tumor ablation techniques, such as radiotherapy, cryoablation and high-intensity focused ultrasound (HIFU), are successfully applied for the treatment of an array of malignancies. HIFU is a completely noninvasive ablation therapy, which uses a multi-element ultrasound transducer to focus high intensity ultrasound beams to a small focal zone. The convergence of acoustic waves results in delivery of acoustic energy to a well-defined region without damaging pre- and post-focal tissue structures. Depending on how the ultrasound is applied, ultrasound energy deposition has either thermal or mechanical effects. Thermal HIFU (T-HIFU) elevates tissue temperature at the focal zone to 60-85°C, resulting in protein denaturation and local coagulative necrosis. Heat diffusion leads to a temperature gradient outside the focal area, where cells are exposed to sublethal temperatures and either undergo apoptotic cell death or recover from reversible injury (1). Mechanical (M)-HIFU generates mechanical damage as result of acoustic cavitation (2–4). The high-pressure waves produce changes in the gaseous components in tissues, as gas bubbles will start to oscillate and burst, causing mechanical damage at a subcellular level (5). Previously, murine studies have demonstrated that M-HIFU can produce completely fragmented tumor lesions with only minor temperature increases (<6°C) (1, 6). The created lesion is homogeneous, with no visible cellular components, and has a sharp demarcated border between damaged and vital tumor tissue (1). Image guidance is used to plan, monitor and evaluate ablation therapy ensuring accurate ablation of tumors while nearby healthy tissues are spared (7). Ideally, magnetic resonance (MR) imaging guidance is used because of its excellent soft-tissue contrast multiplanar imaging, thermometry and motion compensation strategies (7, 8).

Despite their differences, T-HIFU and M-HIFU both create availability of ablated tumor material *in situ*. Tumor debris is a potential tumor antigen source for the induction of anti-tumor immunity (9). Dendritic cells (DCs), the most potent antigen-presenting cells, are able to scavenge tumor debris and present tumor antigens to T cells in draining lymph nodes (LNs). Alternatively, tumor antigens are transported to LNs through lymphatic drainage and presented by LN-resident DCs. Importantly, the context in which DCs capture tumor antigens, such as the cytokine and chemokine environment as well as other endogenous signals, is crucial for initiation and direction of adaptive immunity (10). Tumor ablation releases various bioactive molecules, including danger-associated molecular patterns (DAMPs), contributing to DC maturation (11). Although many studies describe the release of DAMPs or other immunological mediators after HIFU ablation [as reviewed in (12)], the exact immunological correlates that help building strong anti-tumor immune responses are poorly defined. Next to the immunostimulatory effects, a wound healing response is initiated, maintaining immunological tolerance toward the damaged tissue. The presence of immunosuppressive cytokines, such as transforming growth factor (TGF)-β and interleukin (IL)-10, impairs DC functionality and can easily overshadow the ablation-induced immune activating signals (13–15). It is currently unknown how different HIFU ablation strategies influence the (immunological) contexture of the antigen depot.

Although preliminary data suggest that immunomodulating effects occur after HIFU ablation (16–19), no potent tumor-specific immunity has yet been convincingly demonstrated (12). In general, anti-tumor immune responses following *in situ* tumor ablation remain weak without co-exposure to potent immune stimulatory signals. This strengthens the notion that ablation should be combined with immunomodulatory adjuvants to boost anti-tumor immunity (12, 20, 21). For cryoablation, it has been reported that adjuvant administration [in these studies TLR9 agonist cytidyl guanosyl (CpG)] immediately after ablation improved anti-tumor T cell responses (22, 23). Adjuvant administration increased the involvement of mature LN DCs actively scavenging and cross-presenting antigens from the tumor depot (24). It is currently unknown if HIFU conditions exist that induce robust T cell responses, and to what extend they synergize with immune stimulating therapies, such as adjuvants.

In this study, we investigated the immunological effects of MR-guided T- and M-HIFU ablation using the murine ovalbumin-expressing lymphoma model EG7 and explored the potential of HIFU ablation plus CpG adjuvant administration. We report that M-HIFU monotherapy enhanced DC maturation in the tumor-draining LN (TdLN). Administration of CpG following both T- and M-HIFU ablation increased DC maturation in TdLNs relative to mock treatment plus CpG administration to a similar extent. Interestingly, only *ex vivo* co-cultures using TdLN cells from M-HIFU plus CpG treated mice potently induced OT-I T cell proliferation. In search for the potential underlying mechanisms causing this effect, we characterized tumor debris generated by both HIFU methods. M-HIFU debris displayed clear signs of RNA, DNA and protein fragmentation. In addition, western blot analysis showed that protein levels of the immunosuppressive cytokines of TGF-β and IL-10 were significantly reduced. Collectively, these results suggest that M-HIFU ablation induces a unique molecular contexture of the ablated tumor material, resulting in enhanced T cell responses when combined with CpG.

## Materials and methods

### Animal and tumor models

Female C57BL/6NCrl and B6.SJL-*Ptprc^a^Pepc^b^*/BoyJ (8-12 weeks old) mice were purchased from Charles River (respectively from Salzburg, Germany and Calco, Italy). The B6.SJL-*Ptprc^a^Pepc^b^*/BoyJ mouse strain carries the differential pan-leukocyte marker CD45.1. C57Bl/6 OT-I × CD90.1^+^ (Thy-1.1) mice that produce CD90.1^+^CD8^+^ T cells with a transgenic T cell receptor specific for the chicken ovalbumin (OVA) epitope SIINFEKL (OVA_257-264_) presented on MHCI H-2k^b^ were bred and held in the Nijmegen animal facility. Drinking water and standard laboratory food pellets were provided *ad libitum* and mice were allowed to settle for at least 1 week before the start of the experiment. All mice were maintained under specific pathogen-free barrier conditions at the Central Animal Laboratory (Radboudumc, Nijmegen, The Netherlands). All experiments were approved by the Animal Experiment Committee of the Radboud University Medical Center (Radboudumc, Nijmegen, The Netherlands) and performed according to institutional, national and European guidelines.

The EG.7-OVA lymphoma cell line (EG7) is derived from transfection of EL4 cells with a plasmid carrying OVA and G418 resistance gene. EG7 cells were cultured in RPMI medium, supplemented with 10% heat-inactivated fetal bovine serum (FBS, Greiner Bio-One), 2 mM L-glutamine (Lonza, Walkersville, MD), 1% antibiotic–antimycotic solution (100 U/mL penicillin G sodium and 100 μg/mL streptomycin, Gibco) and 50 μM β-mercaptoethanol supplemented with 0,5 mg/ml G418 (Gibco). For tumor cell injection, cells were suspended in phosphate buffered saline (PBS) and injected subcutaneously at the right femur (0.5 × 10^6^ cells). Viability of tumor cells before injection exceeded 95% (determined by trypan blue staining). Tumor growth was evaluated daily with calipers. When the tumor reached 7-9 mm in diameter (after 9-12 days), the mice were randomized and HIFU ablation was performed as described below.

### Adjuvants, reagents and antibodies

CpG-ODN 1668 (‘5-TCCATGACGTTCCTGATGCT-3’) with total phosphorothioate-modified backbone was purchased from Sigma Genosys (Haverhill, UK). OVA_257-264_ (SIINFEKL) was obtained from AnaSpec Inc. (San Jose, CA). The dye carboxyfluorescein succinimidyl ester (CFSE) was purchased from Thermo Scientific. Nancy-520 was obtained from Sigma-Aldrich. For western blot the following antibodies were used: polyclonal anti-TGF-β from Cell Signaling Technology, polyclonal anti-IL-10 from R&D systems and anti-actin (AC-40) from Sigma-Aldrich. For flow cytometry experiments the following antibodies conjugated to various fluorophores were used: anti-Ly6G-FITC (1A8), anti-B220-FITC (RA3-6B2), were purchased from Antibodychain and anti-CD86-PE (GL1) from eBioscience, Inc. (San Diego, CA). Anti-MHCII-BV510 (M5/114.15.2) and anti-CD90.1-APCCy7 (OX-7) were purchased from Biolegend. Purified anti-CD16/CD32 (2.42 G), anti-CD11c-BUV395 (N418), anti-CD80-BV785 (16-10A1), anti-PD-L1-APC (MIH5), anti-CD8α-BV450 (53-6.7) were purchased from BD Biosciences (BD Pharmingen).

### HIFU ablation treatment set-up and protocol

The treatment and imaging set-up employed in this study was extensively described elsewhere (1, 25). Minor adjustments have been made to the existing HIFU system to adapt the system to a smaller bore size due to a different gradient insert (114 mm bore) in the MR magnet. A detailed overview of the HIFU set-up is provided in **Figure S1**.

Mice were anesthetized by 3% isoflurane gas inhalation and maintained by 1–2% isoflurane during treatment to keep the breathing frequency between 30 and 60 per minute. For acoustic coupling, the tumor area was shaven and, if necessary, remaining hair was removed with standard hair removal cream. Breathing rate and body temperature were monitored during the entire procedure with, respectively, a pneumatic balloon and an optical rectal thermometer. Body temperature was controlled with warm air. The mouse was positioned in a right lateral position with the tumor facing downwards inside a degassed water bath on top of the MR compatible animal HIFU system (Image Guided Therapy (IGT), Pessac, France). A 3 MHz HIFU transducer (16 channels annular array cylindrical transducer, diameter 48 mm, 86.58° aperture, adjustable focus depth 30–80 mm) with a full-width-half maximum focal spot of 0.5 × 0.5 × 2.0 mm was used for treatment. Trajectory planning software (Thermoguide, IGT, Pessac, France) was used for treatment planning and guidance.

The mice were randomized at initiation of the experiment and were divided into three groups: 1) non-HIFU treated controls (mock), 2) thermal HIFU (T-HIFU) or 3) mechanical HIFU (M-HIFU). Mock animals were anesthetized and positioned in the HIFU system for MR imaging, without acoustic output by the transducer. Potential confounding was minimized by performing HIFU and mock treatments in a random order, also mice from different treatment groups were randomly co-housed. All animal experiments were conducted in a non-blinded fashion. Mice with tumor ulceration were excluded from the analysis.

The M-HIFU settings used for treatment of the tumors is similar as previously described (1). A custom ablation trajectory was designed to limit local temperature rise: each focal spot received one pulse (5 ms with an acoustic output power of 155–160 W, total acoustic dose 77.5-80 J per sonication), with a pause of 495 ms before moving to the next spot. After each spot received one pulse, the trajectory was repeated for a total of 100 pulses per focal spot. Median 23 (range 13 – 50) focal spots were applied per tumor, 1 mm apart. Animals of the T-HIFU group were treated with 4 s continuous wave sonication, with an acoustic output of 35-37 W (total acoustic dose 140-148 J per sonication). Due to thermal diffusion a slightly larger area was affected per sonication. Therefore, median 17 (range 6 – 25) focal spots were applied per tumor, 1.5 mm apart, with a 40 s cooling time in between sonication. Focal spots were applied in one or two planes, depending on the thickness of the tumor. We aimed to treat a similar percentage of the tumor (approximately 20-30%) with T-HIFU and M-HIFU. The HIFU set-up was positioned in a 7T small animal MR system (ClinScan, Bruker Biospin, Ettlingen, Germany) for treatment planning, evaluation and real-time thermometry.

### MR imaging

MR imaging was performed on a 7T small animal MR system using a homebuilt transmit-receive surface coil (diameter 42 mm). Before and immediately after treatment, a series of T2-weighted MR images was acquired (Turbo Spin Echo (TSE) sequence, repetition time (TR) = 2500 ms, echo time (TE) = 25 ms, Field Of View (FOV) = 45 mm, matrix = 256 × 256, Slice Thickness (ST) = 1 mm, 16 slices), in three orthogonal planes (sagittal, transversal and coronal). For real-time MR thermometry guidance, a T1 weighted Echo Planar Imaging (T1w-EPI) sequence based on a proton resonance frequency shift method was used (TR = 20.91 ms, TE = 4.50 ms, Flip Angle (FA) = 15, FOV = 35 mm, matrix = 64 × 60, ST = 1 mm, 3 slices and total acquisition time (TA) = 1.8 s for T-HIFU and 6 slices and TA = 4.6 s for M-HIFU). Detailed parameters of the MR sequences have been described previously (1). Difussion weighted (DW) imaging was acquired with a DW EPI sequence (TR = 2000 ms, TE = 41 ms, FOV = 45 mm, matrix = 184 × 184, ST = 1 mm, 16 slices, 4 b-values of 0, 100, 500 and 1000 s/mm^2^).

### Tumor debris characterization

*RNA isolation.* Total RNA was isolated from tumor debris using TRIzol reagent (Invitrogen Life Technologies) according to the manufacturer’s instructions, with minor modifications. RNA quantity was determined on a NanoDrop spectrophotometer and 1.5 µg of RNA was loaded on a 1% agarose gel, and after gel electrophoresis stained with Nancy-520. Gel images were acquired and analyzed with the ChemiDoc XRS system (Biorad).

*DNA isolation.* Total DNA was isolated from tumor debris using the NucleoSpin Tissue kit (Macherey-Nagel, Düren, Germany) according to the manufacturer’s instructions. Tumor pieces were lysed in 2 hours, while tumor debris lysates and cell lines were lysed in 15 min. DNA quantity was determined on a NanoDrop spectrophotometer and 1 µg of DNA was loaded. DNA gel electrophoresis was performed using 1.5% agarose gel. Following Nancy-520 staining, images of the gel were captured with the ChemiDoc XRS system.

*Protein isolation for Coomassie blue staining.* Protein extraction using TRIzol reagent proceeded according to the manufacturer’s instruction. Pellets were resuspended in lysis buffer consisting of 1% SDS and 62.5 mM Tris (pH 6.8) plus protease inhibitor cocktail (PIC, cOmplete, Roche) and heated to 50°C. Subsequently, sample buffer (4x concentrated mix: 20% glycerol, 6% sodium dodecyl sulfate (SDS), 125 mM Tris-HCL (pH 6.8), 0.1 mg/ml bromophenol blue (Santa Cruz) and 10% β-mercaptoethanol (Sigma)) was added to samples (1x final concentration) and the suspension was heated to 95°C. Next, equal volume of protein sample was loaded on a 10% bis-acrylamide SDS-PAGE gel. The separated proteins were visualized by Coomassie Brilliant Blue (Bio-Rad) staining.

*Protein isolation for Western Blot.* Control tissue and tumor debris were resuspended in lysis buffer containing 1% SDS and 62.5 mM Tris (pH 6.8) plus PIC and 1 mM phenylmethylsulfonyl fluoride (PMSF, Sigma). Samples were 10-15 times passed through a 25G needle (BD) to homogenize lysates. Afterwards, samples were centrifuged at 20.000 × *g* for 20 min. Protein concentrations were normalized using a bicinchonic assay (BCA, Thermofisher Scientific) to 100 µg/lane or 150 µg/lane, depending on the blot. Lysates were mixed with sample buffer, heated at 95°C for 5 min; and then cooled on ice. Proteins were separated with a 12% bis-acrylamide SDS-PAGE gel. Proteins were transferred to Amersham Protran Nitrocellulose membranes (Sigma). Equal protein loading and transfer on the membrane was checked using a Ponceaus S staining (Sigma). To prevent nonspecific protein binding, the membranes were incubated with 5% milk in Tris-buffered saline (TBS) supplied with 0.1% tween (TBS-T) for 1 h (TGF-β and actin) or 2 h (IL-10). The membranes were then washed 3 times 5 min using TBS-T and subsequently incubated with primary antibody (anti-TGF-β 1:1000, anti-IL-10 0,1 µg/ml, anti-actin 1:5000) overnight at 4°C. Next, the membranes were washed 3 times 5 min using TBS-T, followed by incubation with secondary antibodies (Goat-anti-Rabbit IRD 680RD, Donkey-anti-Rabbit IRD 680RD or Donkey-anti-Goat IRD 800 CW (Licor) all 1:5000) for 1h at RT. All membranes were then washed three times 5 min in TBS-T. After staining, the membranes were scanned using the Oddysey CLx (Licor) to visualize the proteins. Quantification of western blot bands was performed using Image Studio Software (Licor).

### Read-outs

*DC maturation experiment.* CD45.1^+^ mice were subjected to mock, T-HIFU or M-HIFU treatment as described above. Mice subjected to HIFU treatment plus immune activation were injected with 50 µg CpG 1668 in 50 µL PBS within 30 minutes after HIFU ablation, divided over two injections in the peri-tumoral area. Mice subjected to HIFU treatment without immune activation received 50 µL PBS. Two days after HIFU treatment, mice were sacrificed by cervical dislocation followed by excision of both TdLNs and non-draining inguinal LNs. LN were processed into single cells suspension. Hereto, murine LNs were incubated in serum-free RPMI medium containing 1 mg/mL collagenase Type III (Worthington) and 30 μg/mL DNAse type I (Roche) for 30 min at 37°C. EDTA was added to a final concentration of 1 mM and single-cell suspensions were made by passing the cells over a 100 μm cell strainers (Corning). Single cell suspensions were incubated with anti-Fc receptor antibody (clone 2.4G2, BD Biosciences) for 10 minutes on ice. For extracellular staining, the cells were stained with the specific antibodies in PBA (1× PBS, 1% BSA, and 0.02% sodium azide) for 30 minutes in the dark on ice. Cells were washed twice with PBA and measured on a Cytoflex LX Flow cytometer (Beckman Coulter, Fullerton, CA) and data were analyzed using FlowJo software (Tree Star Inc., Ashland, OR). Gating strategy for migratory and resident DCs in LNs is provided in **Figure S2**.

*Ex vivo OT-I co-culture experiment.* Mice were subjected to mock, T-HIFU or M-HIFU treatment as described above. Within 30 minutes after the HIFU treatment, the mice received a peritumoral injection of 50 µg of CpG in 50 µL PBS, divided over two injections in the peri-tumoral area. 16-24 hours after HIFU treatment, mice were sacrificed by cervical dislocation followed by excision of inguinal LNs. LN were processed into single cells suspension using incubation in serum-free RPMI medium containing 1 mg/mL collagenase Type III and 30 μg/mL DNAse type I for 30 min at 37°C. EDTA was added to a final concentration of 1 mM and cell suspension was passed over a 100 μm cell strainer. Concurrently, spleens were harvested from OT-I × CD90.1^+^ mice and mashed over a 100 µM filter to obtain a single cell suspension. CD8^+^ OT-I cells were isolated with EasySep Mouse CD8^+^ T cell isolation kit (StemCell Technologies) and CFSE-labeled according to manufacturer’s protocol (3 μM, Thermo Scientific). Subsequently, 0.75 × 10^6^ LN cells from mock and HIFU treated mice were co-cultured with 0.5 × 10^5^ CFSE-labeled CD8^+^ OT-I T cells for 72 h. As positive control, LN cells were pulsed with 5 ng/mL OVA K^b^ peptide. For the readout of OT-I proliferation, cells were stained extracellularly for CD90.1 and CD8α. CFSE dilution of CD8α^+^CD90.1^+^ cells was measured on a FACSCanto™ II system (BD Biosciences) and data were analyzed using FlowJo software.

*Tumor debris characterization.* Immediately after treatment, mice were sacrificed by cervical dislocation for tumor debris extraction. M-HIFU tumor debris was extracted using a 20G needle (BD) which was inserted into the tumor to aspirate tumor debris. Thermally ablated tumors were excised from mice and the ablated area, visible as white tissue, was cut out using a scalpel (Dahlhausen). Tumor samples were snap-frozen in liquid nitrogen immediately after extraction to allow batch processing. RNA, DNA and protein content was isolated and characterized as described in ‘tumor debris characterization’.

### Statistical analysis

Statistical analyses were performed using Prism 9 software (GraphPad Software Inc.). Sample sizes were calculated using previously published ablation data on DC maturation and T cell activation as primary outcome measures (24, 26). Differences in DC surface expression of activation markers and in OT-I proliferation between treatments were analyzed using an ordinary two-way ANOVA followed by Tukey’s multiple comparison test. Differences between LNs were analyzed using repeated measures two-way ANOVA followed by Bonferroni’s multiple comparison test. Differences in protein level/intensity on western blot were analyzed using repeated measures two-way ANOVA using Bonferroni’s multiple comparison test. Differences were considered significant when P values were smaller than 0.05. In all figures, results are expressed as mean values with standard deviation (SD), while the following symbols were used: p < 0.05 *, p < 0.01 **, p < 0.001 ***, p < 0.0001 ****.

## Results

### MR-guided thermal and mechanical HIFU treatment

**Figure 1:**
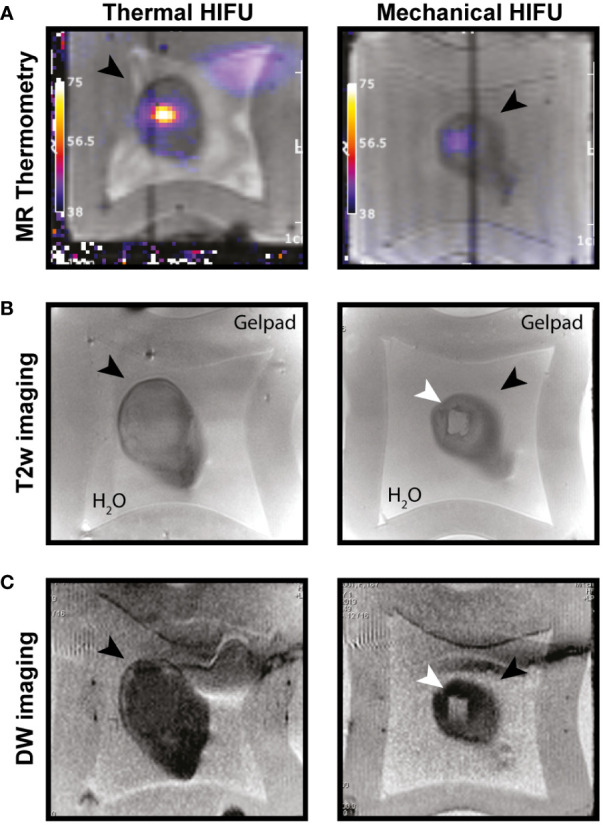
MR imaging of T-HIFU and M-HIFU ablation. Coronal MR images of EG7 tumors during **(A)** and immediately after **(B, C)** thermal (T-) and mechanical (M-)HIFU ablation (tumor, depicted by black arrowhead, is surrounded by water and a gelpad). **(A)** T1 weighted image with temperature map overlay during T-HIFU (left) or M-HIFU (right) ablation. Temperature map shows high temperature increase during T-HIFU ablation. **(B)** Coronal T2 weighted MR images and **(C)** DW images recorded immediately after T-HIFU (left) or M-HIFU (right) treatment. Both T2 weighted and DW images reveal a hyper-intense lesion after M-HIFU treatment (indicated with white arrowhead). DW: diffusion weighted; HIFU, high intensity focused ultrasound; MR, magnetic resonance; T2w, T2 weighted.

Previously we established optimal conditions for HIFU ablation of EL4 thymoma tumors, growing subcutaneously at the hind leg of mice, by either M-HIFU (100 pulses of 5 ms per focal spot) or T-HIFU (4 s continuous wave) (6). In this study, these settings were used for M-HIFU and T-HIFU treatment of EG7 tumors to investigate the immunomodulatory effects following HIFU. Hereto, mice carrying a tumor of 7-9 mm in diameter were treated with T-HIFU or M-HIFU ablation. HIFU treatment was successful in all animals and they recovered well from anesthesia following HIFU ablation. After M-HIFU treatment, no damage was detected in surrounding tissues, while after T-HIFU treatment minor complications occurred (i.e. difficulty in using the tumor bearing leg n = 3/13, and redness of the skin n = 2/13). MR thermometry showed peak temperatures during treatment of 78.8°C (SD. 6.4) and 40.7°C (SD. 0.8) for T-HIFU and M-HIFU, respectively, as previously reported (**Figure 1A**) (25). T2w MR imaging revealed a clear hyper-intense region, which correlated with the planned treatment area (**Figure 1B**). As an alternative approach, we investigated the ability of diffusion weighted (DW) imaging for HIFU treatment evaluation. Immediately after M-HIFU treatment, DW images revealed a clear hyper-intense area, which correlated with the T2w MR images (**Figure 1C**). For T-HIFU, limited signal changes were visible on T2w MR imaging as well as DW imaging immediately after treatment (**Figures 1B, C**).

### M-HIFU increases maturation and activation of TdLN dendritic cells

**Figure 2:**
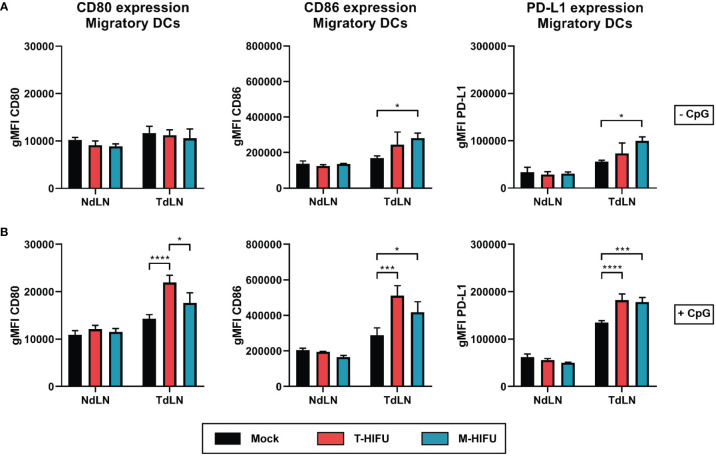
Migratory DC maturation following HIFU ablation. **(A, B)** Mice bearing EG7 tumors were treated with mock, T-HIFU or M-HIFU ablation **(A)**. Mice subjected to HIFU treatment plus immune activation **(B)** were injected peritumorally with 50 µg CpG within 30 minutes after HIFU ablation. Two days after HIFU treatment, inguinal LNs were excised and analyzed for DC maturation using flow cytometry. Bar graphs show geometric mean fluorescent intensity (gMFI) ± SD of CD80 (left), CD86 (middle) and PD-L1 (right) on CD11c^+^MHCII^high^ migratory LN DCs without **(A)** or with CpG administration **(B)**. Statistical significance was calculated using an ordinary two-way ANOVA followed by Tukey’s multiple comparison tests (mock minus CpG n = 2, other groups n = 3 animals per group). CpG, cytidyl guanosyl; DC, dendritic cell; gMFI, geometric mean fluorescent intensity; M-HIFU, mechanical-high intensity focused ultrasound; NdLN, non-draining lymph node; PD-L1, programmed death-ligand 1; T-HIFU, thermal-high intensity focused ultrasound; TdLN, tumor-draining lymph node. p < 0.05 *, p < 0.001 ***, p < 0.0001 ****.

The tumor debris generated by tumor ablation comprises of tumor antigens that can be scavenged by immune cells, like professional antigen-presenting DCs, to mount anti-tumor immune responses. As previously shown, cryoablation results in increased maturation of DCs (24). Here, we set out to explore the effect of HIFU ablation methods on LN DC activation and functionality. Mice bearing EG7 tumors were treated with mock, T-HIFU or M-HIFU and two days following HIFU treatment, DC maturation in LNs was assessed. We observed that M-HIFU significantly increased the expression of the co-stimulatory molecule CD86 as well as the activation marker PD-L1 on CD11c^+^MHCII^high^ migratory DCs in the TdLN compared to mock mice (**Figure 2A**). In contrast, T-HIFU slightly, but not significantly, increased the expression of CD86 and PD-L1 on migratory DCs in the TdLN compared to mock treated mice (**Figure 2A**). Expression of CD80 was not altered upon M-HIFU or T-HIFU ablation (**Figure 2A**). No difference in activation marker expression was observed in non-draining LN (NdLN) DCs upon HIFU ablation (**Figure 2**). In general, maturation markers were more evidently increased on CD11c^+^MHCII^high^ migratory DCs compared to CD11c^+^MHCII^int^ resident DCs (**Figure S3**).

DC maturation was also assessed after peritumoral injection of the TLR9 agonist CpG. CpG is an adjuvant that potently induces DC maturation including the upregulation of co-stimulatory molecules and was previously shown to act synergistically with cryoablation to enhance the maturation state of TdLN DCs (24). CpG injection alone increased expression of CD80 (geometric mean fluorescent intensity (gMFI) 1.23-fold), CD86 (gMFI 1.71-fold) and PD-L1 (gMFI 2.41-fold) on migratory DCs in TdLNs (**Figure 2**). T-HIFU with CpG co-administration significantly enhanced expression of CD80, CD86 and PD-L1 on CD11c^+^MHCII^high^ migratory DCs compared to mock plus CpG administration (**Figure 2B**). M-HIFU ablation combined with CpG also significantly augmented CD86 and PD-L1 expression on migratory DCs compared to mock treatment. The same trend was observed for CD80 expression following M-HIFU and CpG treatment, but the increase was much less as compared to T-HIFU treatment with CpG (**Figure 2B**). Altogether, these results indicate that M-HIFU alone enhanced DC maturation in TdLNs. CpG administration further increased DC maturation following M-HIFU as well as T-HIFU ablation.

### M-HIFU plus CpG administration enhances dendritic cell-induced T cell activation *ex vivo*

**Figure 3:**
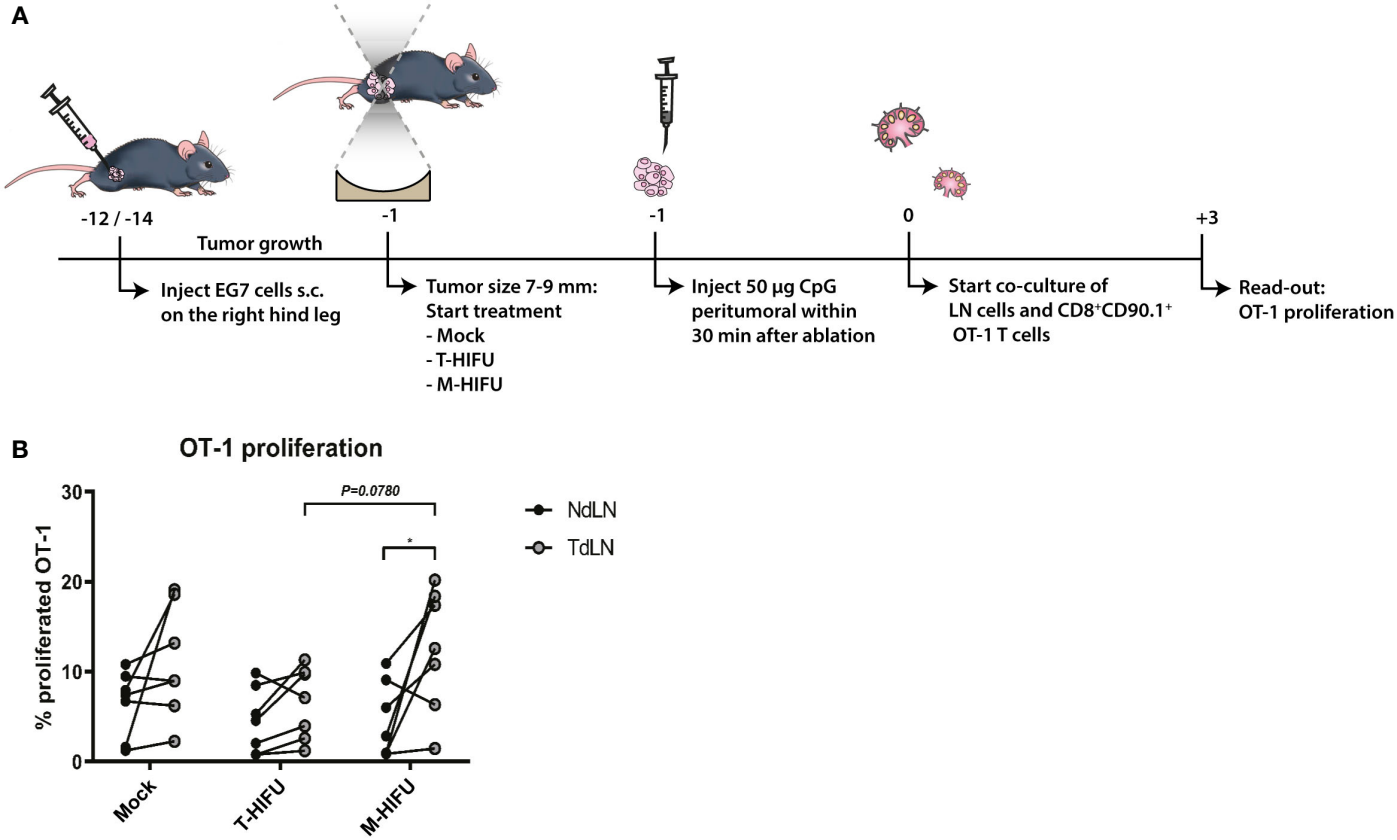
*Ex vivo* OT-I proliferation following T-HIFU or M-HIFU. **(A)** Diagram shows the treatment schedule for assessing OT-I proliferation following HIFU ablation. Mice bearing EG7 tumors were treated with mock, T-HIFU or M-HIFU ablation and received a peritumoral injection of 50 µg CpG within 30 minutes after HIFU ablation, divided over two injections. 16-24 hours after HIFU treatment, inguinal LNs were excised and single cell LN suspensions (0.75 × 10^6^ cells) were co-cultured with 0.5 × 10^5^ CFSE labelled CD8^+^ OT-I T cells for 72 h. **(B)** Graph shows OT-I proliferation upon co-culture of NdLN and TdLN cells following mock, T-HIFU and M-HIFU treatment plus CpG administration. Differences in OT-I proliferation between treatments were analyzed using ordinary two-way ANOVA followed by Tukey’s multiple comparison test. Differences between LNs were analyzed using repeated measures two-way ANOVA followed by Bonferroni’s multiple comparison test (control n = 7, T-HIFU n = 7, M-HIFU n = 7 animals). Shown are pooled results from three independent experiments. CFSE, carboxyfluorescein succinimidyl ester; CpG, cytidyl guanosyl; LN, lymph node; M-HIFU, mechanical-high intensity focused ultrasound; NdLN, non-draining lymph node; T-HIFU, thermal-high intensity focused ultrasound; TdLN, tumor-draining lymph node.

To assess DC function following HIFU ablation plus CpG administration, we determined the ability of DCs to cross-prime OVA-specific CD8^+^ OT-I T cells. To this end, mice bearing OVA expressing EG7 tumors were mock-treated or treated with T-HIFU or M-HIFU and injected with CpG peritumorally within 30 min after ablation. One day after ablation, inguinal TdLN and NdLN were harvested, processed into single cells suspension, and subsequently co-cultured with CD8^+^ OT-I T cells (**Figure 3A**). There was no significant difference in the proliferation of OT-I T cells when comparing TdLN and NdLN cells in co-cultures derived from mock or T-HIFU mice co-treated with CpG (**Figure 3B**). However, a significant increase in OT-I proliferation was found in co-cultures with TdLN cells compared to NdLN cells derived from M-HIFU treated mice receiving CpG (**Figure 3B**). Besides, the percentage of proliferated OT-I T cells co-cultured with TdLN cells from M-HIFU treated mice was slightly elevated in comparison to T-HIFU treated mice. These results indicate that TdLN cells derived from M-HIFU, rather than T-HIFU, most effectively cross-present antigen to CD8^+^ OT-I T cells *ex vivo.*

### M-HIFU creates unique tumor debris and reduces immunosuppressive factors

**Figure 4:**
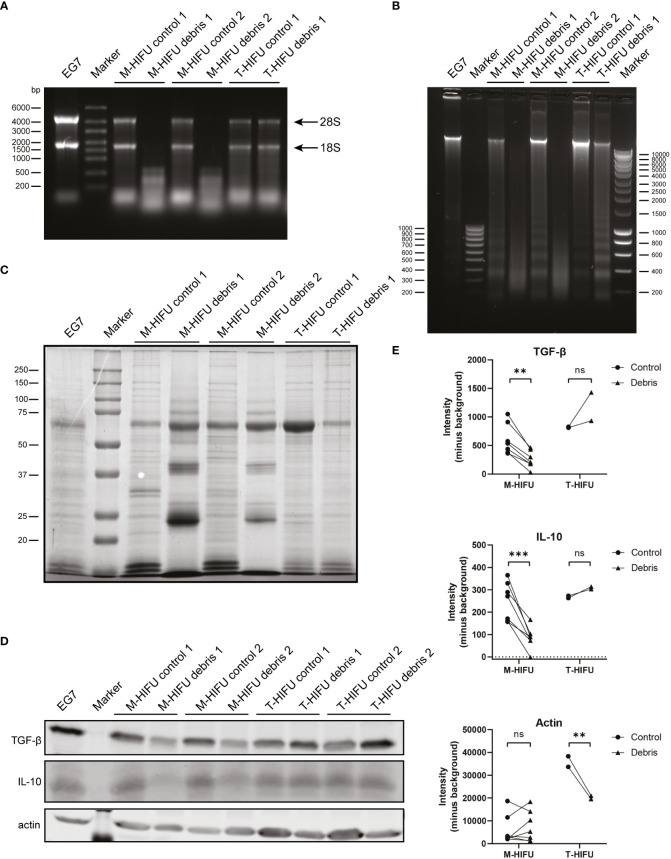
T-HIFU and M-HIFU tumor debris characterization. Mice bearing EG7 tumors were treated with T-HIFU or M-HIFU ablation and immediately after treatment tumor debris was extracted. Tumor debris was characterized for RNA **(A)**, DNA **(B)** and protein content **(C)**. Each lane represents tumor debris and matched control sample obtained from an individual mouse. Western blot analysis **(D)** and quantification **(E)** of TGF-β, IL-10 and actin in tumor debris and corresponding surrounding control tissue. Each lane represents tumor debris and matched control sample obtained from an individual mouse. Statistical significance was calculated using a repeated measures two-way ANOVA followed by Bonferroni’s multiple comparison tests (T-HIFU n = 2, M-HIFU n = 7). IL-10: Interleukin-10; M-HIFU: mechanical-high intensity focused ultrasound; T-HIFU: thermal-high intensity focused ultrasound; TGF-β: transforming growth factor-beta. p < 0.01 **, p < 0.001 ***.

T- and M-HIFU ablation in combination with CpG administration both resulted in migratory DC maturation. However, only the combination of M-HIFU ablation plus CpG administration potently induced antigen-specific T cell proliferation *ex vivo*. Both HIFU ablation methods led to *in situ* tumor destruction, but the different pulse regimens resulted in completely different local temperatures and ways of cell fragmentation. This prompted us to characterize the tumor debris generated by T-HIFU and M-HIFU on a molecular level by analyzing its RNA, DNA and protein content. Mice treated with T-HIFU or M-HIFU were sacrificed immediately after HIFU treatment and treated tumor tissue (referred to as debris) was extracted as well as tumor tissue from outside the ablation area (referred to as control). Alongside the untreated EG7 tumor tissue, the EG7 cell line was included as an additional control. In these control samples, as well as in the T-HIFU tumor debris, intact 28S and 18S ribosomal RNA (rRNA) bands were readily detected (**Figure 4A**). Interestingly, intact rRNA bands were not observed in the M-HIFU tumor debris; instead, the loaded RNA appeared as an RNA-smear in the low molecular weight range (**Figure 4A**). A similar pattern was observed for DNA content, where only M-HIFU tumor debris showed clearly fragmented DNA with a smeared appearance indicative of random DNA breaks (**Figure 4B**). A typical ladder-like pattern of degraded DNA products, a characteristic of apoptosis, was also observed on agarose gel electrophoresis. This apoptotic ladder was most pronounced in the T-HIFU derived tumor debris (**Figure 4B**). Protein content analysis also revealed an altered protein pattern, indicative of protein fragmentation, specifically in the M-HIFU tumor debris (**Figure 4C**). Together, these results show that M-HIFU uniquely induced a fragmented RNA, DNA and protein profile in tumor debris.

Tumor debris not only consist of tumor (neo)antigens, but also contains immune stimulatory and inhibitory components of the treated tumor. Especially the latter has received attention as blocking immune inhibitory checkpoints or immunosuppressive cytokines have been particularly effective and revolutionized cancer treatment. Our observation that the tumor debris content following M-HIFU is strongly fragmented, prompted us to investigate the effect of M-HIFU and T-HIFU treatment on the levels of the immunosuppressive factors TGF-β and IL-10 in the resulting tumor debris. Hereto, equal amounts of protein as determined by a BCA assay were loaded for western blot analysis. Visualization of total protein using Ponceau S staining confirmed equal protein loading (data not shown). Strikingly, M-HIFU debris contained severely diminished absolute protein levels of TGF-β and IL-10 as compared to untreated control tumor tissue. No decreased TGF-β or IL-10 protein levels were observed in T-HIFU treated areas when compared to control tissue (**Figures 4D, E**). Of note, we observed an increased amount of the cytoskeletal protein actin in control T-HIFU samples compared to T-HIFU debris and the EG7 cell line, which is most likely explained by the small temperature rise in the control tissue adjacent to the treated T-HIFU area (**Figures 4D, E**). Altogether, these data indicate that the fragmented tumor debris generated by M-HIFU ablation is different from the debris following T-HIFU ablation in that it contains a unique molecular contexture. Moreover, M-HIFU lowers the local presence of immunosuppressive factors in the tumor debris and thereby the context in which the immune response is elicited.

## Discussion

In this study we investigated the effects of MR-guided M-HIFU and T-HIFU ablation on tumor destruction and immune cell activation with or without co-administration of CpG. Our results show that M-HIFU ablation alone significantly increased DC activation in TdLN. CpG administration within 30 min following M-HIFU or T-HIFU ablation increased DC activation in TdLNs to a similar extend compared to mock plus CpG treatment. Remarkably, although both HIFU ablation modalities increased DC activation to a similar degree in presence of CpG, only co-cultures with TdLN cells from M-HIFU treated mice potently induced OT-I CD8^+^ T cell proliferation *ex vivo*. Molecular characterization of the tumor debris resulting from the two types of HIFU showed that only M-HIFU uniquely induced a fragmented RNA, DNA and protein profile in tumor debris, reducing the presence of immunosuppressive factors TGF-β and IL-10.

Both HIFU ablation methods effectively destroy tumor tissue and result in a depot of tumor material which remains *in situ.* This antigen depot contains antigens in a (partially) denatured or non-denatured state, depending on the temperature reached in the focal area. The fragmented content of the M-HIFU tumor debris is most likely due to fragmentation by the pulsed high-intensity focused ultrasound waves. However, it is difficult to fully exclude that DNAses, RNAses or proteases participated in this effect. In contrast, we observed an increase in apoptotic DNA fragmentation in T-HIFU debris compared to adjacent tissue, whereas the RNA and protein profile appeared largely unaltered. These results indicate that the molecular contexture of M-HIFU and T-HIFU debris is different.

The context in which DCs take up tumor antigens is crucial for engaging protective adaptive immunity. Our data indicate that M-HIFU ablation attenuates the anti-inflammatory cytokines TGF-β and IL-10 within the tumor debris. The degradation of these anti-inflammatory cytokines likely shifts the tumor debris to a less immunosuppressive contexture. It will be interesting to investigate the effect of M-HIFU on other factors present in the debris, such as endogenous danger signals, immune stimulatory molecules and other immune inhibitory molecules. Very different from M-HIFU, T-HIFU debris consisted of denatured and coagulated tumor tissue. Similar levels of TGF-β and IL-10 were observed in T-HIFU control tissue and debris, but actin levels were elevated in control T-HIFU lysates compared to T-HIFU debris and the EG7 cell line. This increase is possibly prompted by T-HIFU-induced heat diffusion resulting in hyperthermia, which has previously been shown to modulate actin dynamics (27). No difference was observed in the actin levels after M-HIFU ablation. Identification and further understanding of the unique antigenic “fingerprint” induced by various *in situ* tumor ablation techniques, including its immunogenicity, will be crucial to link the tumor antigen depot to the induced immune response, and will help further optimizing *in situ* tumor ablation (immunocombination) therapies.

Tumor debris remaining *in situ* after tumor destruction provides the immune system with an antigen source for the induction of anti-tumor immunity. The primary goal is to obtain tumor antigen-loaded DCs in the TdLN that are properly activated so that they can initiate T cell-mediated immunity. Analysis of the maturation state of DCs following HIFU revealed that CD11c^+^MHCII^high^ migratory DCs in the TdLN expressed significantly higher levels of the maturation and activation markers CD86 and PD-L1 upon M-HIFU ablation, whereas T-HIFU did not significantly alter the expression of these markers on DCs. Although PD-L1 is most known for its dampening effect on anti-tumor immune responses through its interaction with PD-1 on T cells, PD-L1 is also a marker of immune activation and important for proper DC functioning. Interestingly, it was recently reported that cDC1s, which are particularly important for the priming and activation of CD8^+^ T cells, upregulate PD-L1 expression upon antigen uptake (28). The underlying mechanism(s) for the upregulation of activation markers on DCs following M-HIFU, and not T-HIFU, remains to be elucidated, but is likely related to endogenous mediators that are produced upon the diverse HIFU ablation modalities. Although various studies reported the release of danger signals that results in DCs activation following HIFU ablation (11, 29), the immunostimulatory potential of these signals appears to be impaired by protein denaturation due to T-HIFU ablation. Furthermore, the balance between immune stimulatory and immune inhibitory molecules determines immune activation. The immunosuppressive mediators can potentially overshadow the ablation-induced immune activating signals. Interestingly, this is exactly what we observed as M-HIFU alone, but not T-HIFU alone, induced DC maturation. Further, our data showed that M-HIFU creates a unique molecular context of ablated tumor material and reduces immunosuppressive molecules, which may partly explain M-HIFU-induced DC-mediated T cell activation.

M-HIFU alone significantly increased DC maturation in TdLNs. In this study we report that CpG administration following HIFU ablation further increased the activation of migratory DCs in TdLNs. In line with these findings, several other studies have reported promising preclinical results of the therapeutic potential of HIFU and immunotherapy combination therapy (30–34). Interestingly, in our study both T-HIFU and M-HIFU significantly increased activation of migratory DCs relative to mock plus CpG treatment, but only co-cultures with TdLN cells from M-HIFU treated mice potently induced OT-I CD8^+^ T cell proliferation *ex vivo*. These results suggest that M-HIFU ablation generates tumor antigens that allow for more effective presentation by LN cells and priming of tumor antigen specific T cells, which are in part also related to improved drainage of the liquified tumor debris towards TdLNs. It remains to be investigated, however, if M-HIFU plus CpG will induce strong anti-tumor (memory) responses capable of rejecting a tumor rechallenge or can induce an abscopal effect. A previous study demonstrated that amputation of the B16-F10 tumor-bearing leg 2 days after thermal or mechanical HIFU ablation resulted in a decreased metastasis incidence rate (19).

The inability to ablate complete tumors using our HIFU system is a potential limitation of our study. Notably, for radiofrequency ablation it has been observed that complete tumor ablation is crucial for synergy with anti-PD1 immunotherapy (35). On the other hand, Eranki et al. showed that partial M-HIFU ablation in a more immunogenic neuroblastoma tumor model synergized with checkpoint inhibitors (anti-PD-L1 plus anti-CTLA4) and significantly prolonged survival (36). This discrepancy possibly depends on the nature and immunogenicity of the antigens released and/or the immune stimulatory capabilities of the *in situ* tumor destruction technique applied. Information on complete or partial ablation is important to compare ablation strategies from an immunological point of view but also provides crucial insights about additional (immuno)therapy effects that influence the immunological outcome.

Tumor ablation techniques allow for direct *in situ* (neo)-antigen loading of DCs without prior knowledge of tumor antigens or epitopes. The challenge within the field of interventional oncology is to create an *in situ* vaccine that drives systemic immune responses following tumor ablation, robust enough to cause a broad and effective systemic anti-tumor immune response. The results described here imply that especially M-HIFU ablation creates a unique antigen depot and enhances DC-mediated CD8^+^ T cell immunity in combination with CpG. M-HIFU ablation is currently clinically tested (NCT04572633). A better understanding of how HIFU ablation influences anti-tumor immunity will lead to more effective *in situ* vaccination strategies.

## Data availability statement

The raw data supporting the conclusions of this article will be made available by the authors, without undue reservation.

## Ethics statement

The animal study was reviewed and approved by the Animal Experiment Committee of the Radboud University Medical Center (Radboudumc, Nijmegen, Netherlands) and performed according to institutional, national and European guidelines.

## Author contributions

GA conceived the project. RB, VM and GA designed the experiments and RB, VM, FS, TR, MW conducted the experiments. RB, VM and GA analyzed and interpreted the data. AV and ED delivered technical support. RB, VM and GA wrote the manuscript. AH, JF and GA supervised the project. All authors contributed to the article and approved the submitted version.
