# Supplementary figures and images for: Mechanical high-intensity focused ultrasound creates unique tumor debris enhancing dendritic cell-induced T cell activation
Source: Front Immunol. 2022 Dec 7;13:1038347. doi: 10.3389/fimmu.2022.1038347 (PMC9768443; doi:10.3389/fimmu.2022.1038347)

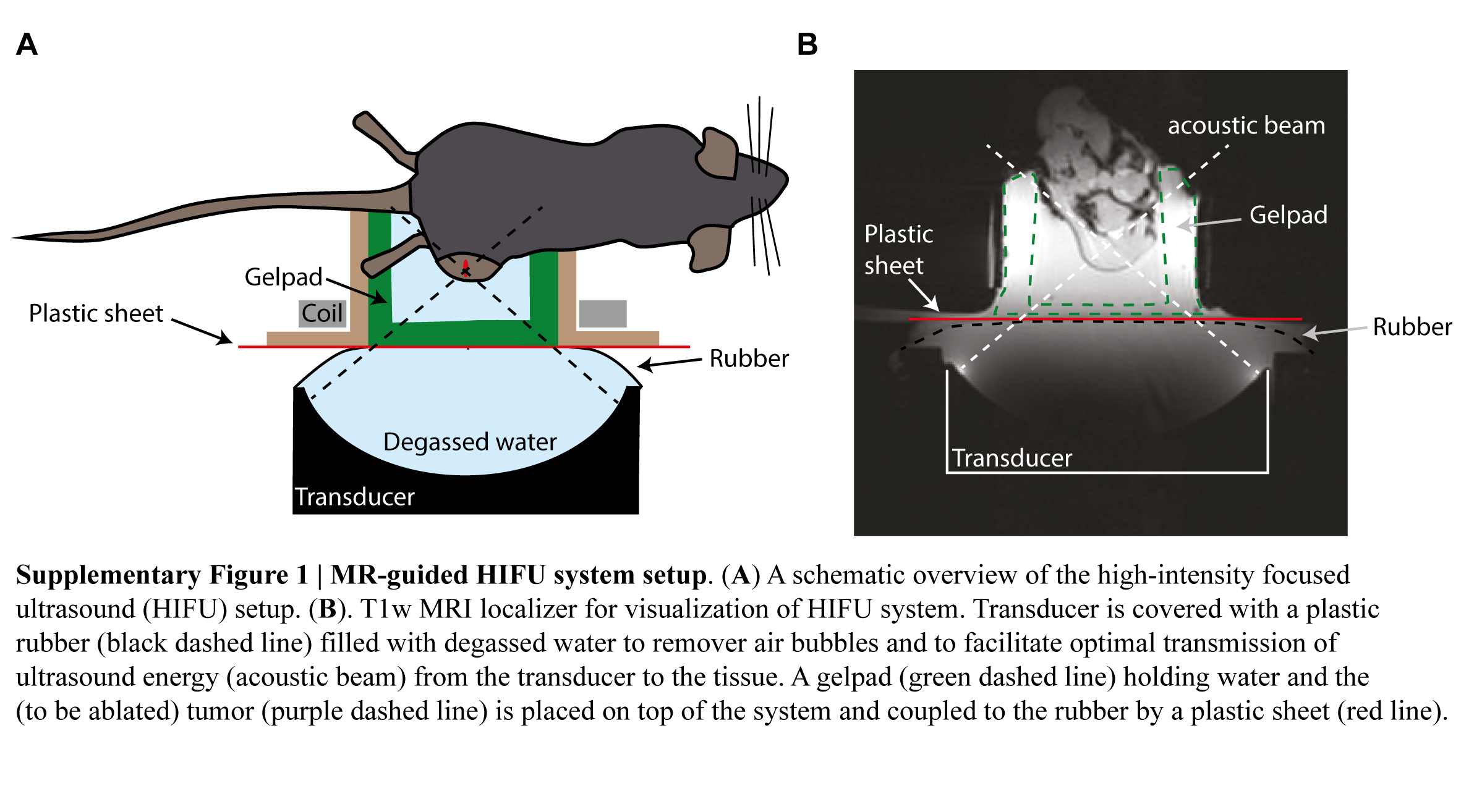

Supplement: Supplementary file 1 [file Image_1.tif]

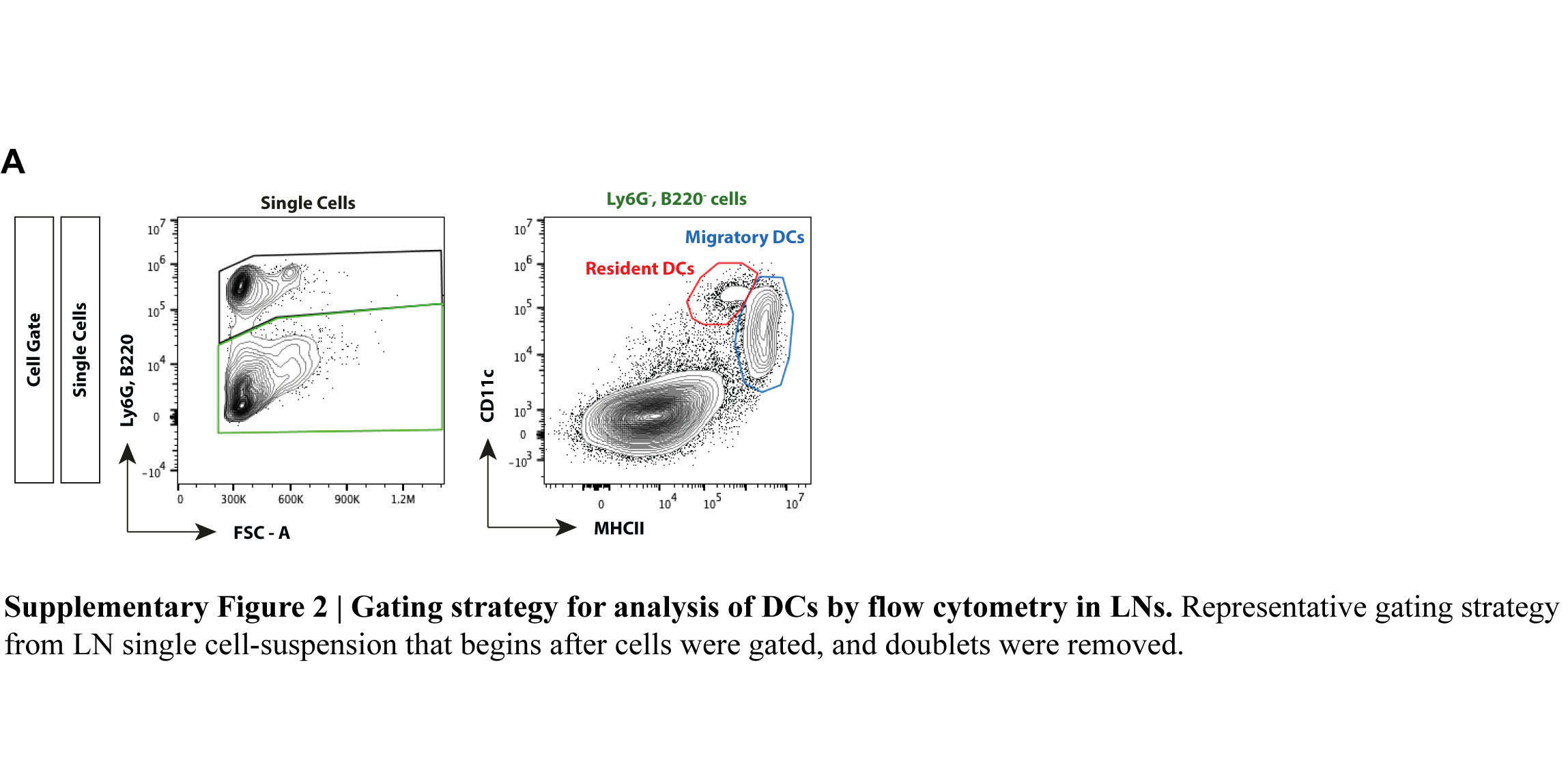

Supplement: Supplementary file 2 [file Image_2.tif]

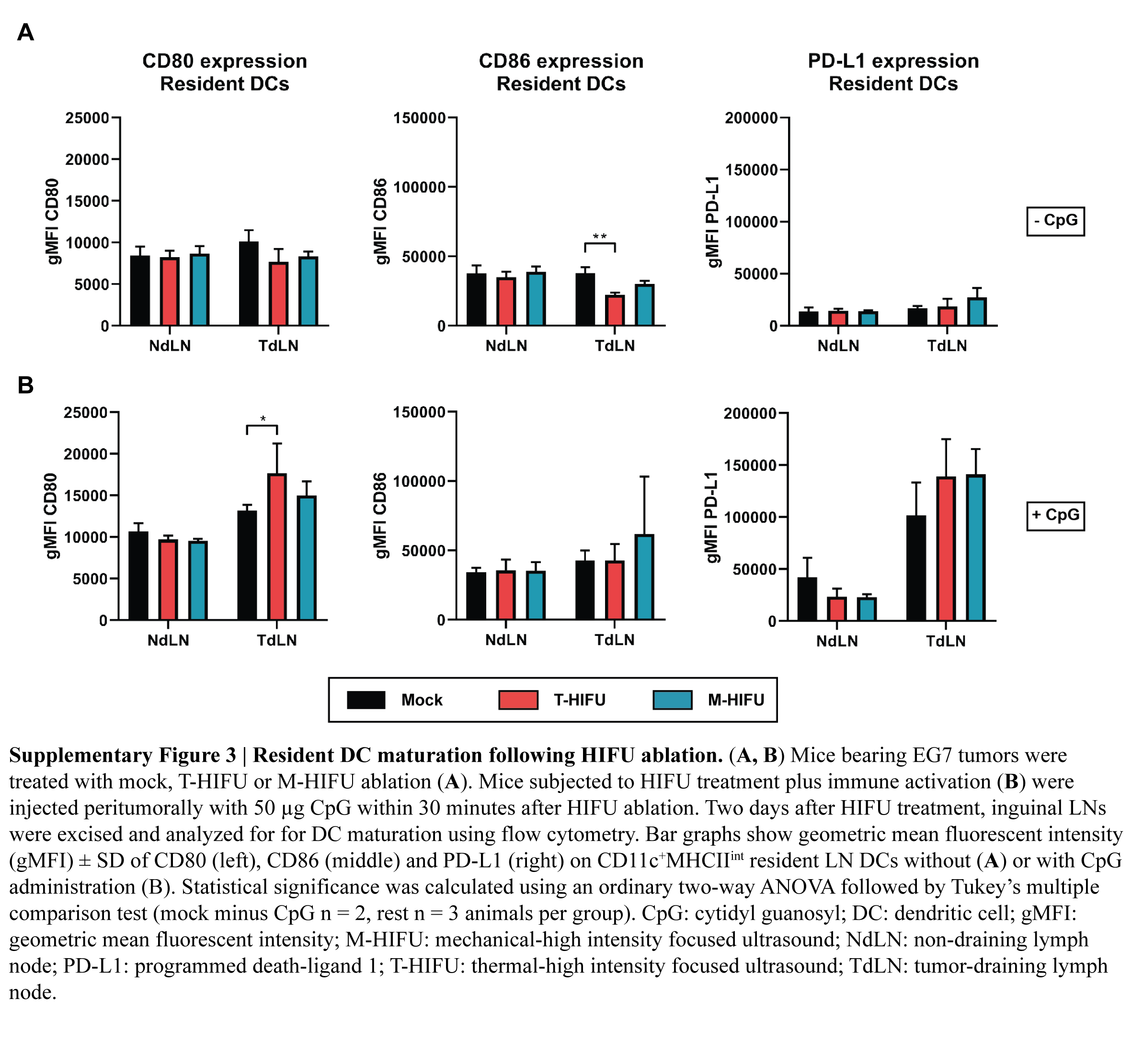

Supplement: Supplementary file 3 [file Image_3.tif]
